# Security of Cryptocurrencies: A View on the State-of-the-Art Research and Current Developments

**DOI:** 10.3390/s23063155

**Published:** 2023-03-15

**Authors:** Paweł Weichbroth, Kacper Wereszko, Helena Anacka, Jolanta Kowal

**Affiliations:** 1 Department of Software Engineering, Faculty of Electronics, Telecommunications and Informatics, Gdańsk University of Technology, Gabriela Narutowicza 11/12, 80-233 Gdańsk, Poland; 2 Department of Algorithms and System Modeling, Faculty of Electronics, Telecommunications and Informatics, Gdańsk University of Technology, Gabriela Narutowicza 11/12, 80-233 Gdańsk, Poland; 3 Faculty of Management and Economics, Gdańsk University of Technology, Gabriela Narutowicza 11/12, 80-233 Gdańsk, Poland; 4 Institute of Psychology, University of Wrocław, Dawida 1, 50-529 Wrocław, Poland

**Keywords:** security, digital currency, cryptocurrency, wallet, architecture, data transmission method, social engineering attack, countermeasures

## Abstract

[Context] The goal of security is to protect digital assets, devices, and services from being disrupted, exploited or stolen by unauthorized users. It is also about having reliable information available at the right time. [Motivation] Since the inception in 2009 of the first cryptocurrency, few studies have been undertaken to analyze and review the state-of-the-art research and current developments with respect to the security of cryptocurrencies. [Purpose] We aim to provide both theoretical and empirical insights into the security landscape, in particular focusing on both technical solutions and human-related facets. [Methodology] We used an integrative review which could help in building science and scholarly research, the basis for conceptual and empirical models. [Results] Successful defense against cyberattacks depends on technical measures on the one hand, as well as on self-education and training with the aim to develop competence, knowledge, skills and social abilities, on the other. [Contribution] Our findings provide a comprehensive review for the major achievements and developments of the recent progress on the security of cryptocurrencies. [Future research] Since there is increasing interest in adoption of the current solutions within the central bank digital currencies, the future research should explore the development and inception of effective measures against social engineering attacks, which still remain the main concern.

## 1. Introduction

The Digital Revolution, also known as the Third Industrial Revolution [1], undoubtedly marks the beginning of the Information Era. The advancement of technology from analog electronic and mechanical devices to digital technology has been remaking the world [2]. This digital revolution has proceeded at breakneck speed since no other human invention has reached more people in as short a space of time as the Internet [3].

The rise of the Internet has changed the way people exchange not only information [4] but also other goods [5], including money. Due to the limitations of local currencies, concerning limited liquidity, proxy transaction costs for foreign payments, and emerging economies’ trust deficit, to name a few, the first cryptocurrency emerged just over a decade ago to overcome these obstacles.

By design, cryptocurrencies (or simply crypto) facilitate peer-to-peer payments without the oversight of an intermediary (such as a bank or any governmental body) [6], and eliminate the need for identification information for both parties [7]. In general, cryptocurrencies and their underlying technology (blockchain) are seen as a source of a radical shift to the “Internet of Value”, which can disrupt the traditional financial world [8]. Despite surging in popularity and being recognized as the most trusted financial instrument by many investors [9], whether crypto will ever go mainstream depends on factors such as price stability, ease of use and security [10].

Indeed, the issue of cybersecurity always brings considerable attention when using cryptocurrencies. According to the Federal Trade Commission, the number of cryptocurrency scams has increased sharply from October 2020 through March 2021, with nearly 7000 people reporting losses totaling more than $80 million. To be clear, the top scams, considering their value and impact, hit business organizations and government bodies. For instance, in February 2014, hackers stole about $460 million in bitcoins from Mt. Gox exchange [11], the world’s largest bitcoin trading exchange with its headquarters in Tokyo. After admitting the 850,000 Bitcoin loss, the exchange was shut down just weeks later, causing the first Bitcoin market crash, as its price slid from $800 to $400 [12]. As we know now, it was not the first organization to suffer a massive theft, and will definitely not be the last.

The ISO/IEC 27032 standard defines cybersecurity as “preservation of confidentiality, integrity and availability of information in the Cyberspace” [13]. In turn, Cyberspace is defined as “the complex environment resulting from the interaction of people, software and services on the Internet by means of technology devices and networks connected to it, which does not exist in any physical form”. Inarguably, with the introduction of cryptocurrencies, new cybersecurity issues have emerged [14,15,16], reshaping and redefining its landscape. Since understanding cybersecurity is no longer optional for businesses and individuals, this study delves into this topic by reviewing and analyzing the state-of-the-art research and current developments.

To the best of our knowledge, few attempts (if any) have been made so far to undertake a similar study. In particular, our study tackles the ongoing discussion on crypto cybersecurity (or simply security) by adopting the grounded theory approach developed by Glaser and Strauss [17], in particular by adapting analytical thinking [18] and sampling strategies [19]. Taking into account the general notion of cybersecurity, in our view, its multidimensional nature considered in the context of cryptocurrencies can be further conceptualized within two mainstream areas, namely: technological and human. In particular, the former concerns four interconnected hardware and software domains, spanning from cryptocurrency wallets to security architectures, models, and data transmission methods. In contrast, the latter considers humans (users) as the last link of the security chain. It should be noted here that the notion of a user is a theoretical lens to consider cybersecurity in terms of social engineering attacks and corresponding countermeasures [20], hence these are also investigated in our study.

While the grounded theory suggests a contextualized understanding of the phenomena [21], we collected, coded and analyzed the data based on an extracted set of keywords (marked in italics). To explore these five topics, we selected and applied guidelines elaborated by Kitchenham and Charters [22]. This methodology, along with its associated principles, has been well received by researchers worldwide, and nowadays is widely adopted not only in the computer science domain. A systematic search was performed on Scopus and Google Scholar using their available online search engines. We also used Google Search to acquire recent market and statistical data. In formulating search queries, we used combinations of the keywords, indicating the relationships between them by specifying explicit logical operators such as AND, OR and NOT [23]. Initially, from the list of the search results, potentially relevant papers were selected, based on an individual evaluation of both the title and abstract. The criteria followed in assessing the quality of a paper were the relevance of its topic, objective and outcome. To ensure that findings were properly classified and synthesized, at least two other authors checked and confirmed their validity.

The rest of the paper is organized as follows. In Section 2, we discuss the background of the development of cryptocurrencies. In Section 3, we define and classify crypto wallets. In Section 4, we review and analyze security architectures, followed by a description and exemplification of the related models given in Section 5. Afterwards, in Section 6, we recognize and localize the data transmission methods developed for blockchain-based solutions. Next, in Section 7, we elaborate on the adopted social engineering attacks and adapted countermeasures for the cryptocurrencies settings. In Section 8, we discuss the findings, including the implications for theory and practice. Eventually, in Section 9, we conclude the paper with a summary of the performed study.

## 2. Background

The Merriam-Webster dictionary defines a cryptocurrency as “any form of currency that only exists digitally, that usually has no central issuing or regulating authority but instead uses a decentralized system to record transactions and manage the issuance of new units, and that relies on cryptography to prevent counterfeiting and fraudulent transactions” [24]. In other words, cryptocurrency is “a digital currency produced by a public network, rather than any government, that uses cryptography to make sure payments are sent and received safely” [25].

There are various ways to mine cryptocurrency:

- Application-Specific Integrated Circuits (ASIC) are a special type of microchip designed to perform a repeated function that hashes blocks in order to find a valid Proof-of-Work [26].
- Central Processing Unit (CPU) utilizes one or more processors and thus is poorly profitable for its users [27].
- A Graphics Processing Unit (GPU) utilizes one or more graphics cards [28] and is currently claimed to be the most popular and well-known method of cryptocurrency mining [29].
- Field-Programmable Gate Array (FPGA) is an electronic circuit that one can program to execute certain logical operations with a programming language such as Verilog or VHDL. FPGAs are more adaptable than ASICs, and faster and more efficient than GPUs [30].

Considering the number of the number of mining participants, the mining process can be performed in two scenarios: individually (solo mining), or in a group (mining pools). Another approach is cloud mining in which computational work from a cloud-computing farm is outsourced. Here, the mining process is easier to implement since it does not require specialized hardware deployed. Nevertheless, cloud computing imposes a number of security issues, including access control, authentication and identification, availability, policy integration, and audit strategies, as well privacy concerns such as unauthorized secondary usage, lack of user control, and unclear responsibility, just to name a few [31].

Although Bitcoin is claimed to be the first established cryptocurrency, there had been preceding attempts at developing digital currencies with ledgers secured by reliable encryption methods. Two examples of these are Bit Gold, invented by Nick Szabo in 1998, and B-Money, introduced by Wei Dai in the same year. However, they were never fully developed and put on the marketplace [32]. Ten years later, on 31 October 2008, the nom de plume Satoshi Nakamoto posted a paper to a cryptography mailing list, entitled “Bitcoin: A Peer-to-Peer Electronic Cash System” [33]. Afterwards, on 11th January 2009, the first bitcoin transaction occurred when Nakamoto sent 10 bitcoins (BTC) to a computer programmer, Hal Finney [34].

On 15 August 2010, one of the most striking security issues in the blockchain network appeared, involving a transaction of 184 billion BTC, well beyond the 21 million supply cap, and 8784 times more than should ever exist [35]. Within five hours of the discovery, Nakamoto released a new version (0.3.1) of the Bitcoin client, with a fix containing a soft fork. As a consequence, two different versions of Bitcoin existed in the immediate hours after version 0.3.1 was published. Eventually, the network made the previously valid blocks that included the exploited transactions invalid. Nineteen hours after the disclosure of the incident, the “good” chain became the dominant one, however the “bad” chain still existed and disrupted some users for at least the next day [36]. Ultimately, the chain introduced in this fixed version became the Bitcoin blockchain that exists today.

Later that year, a programmer named Laszlo Hanyecz bought two pizzas for 10,000 bitcoin at Papa John’s pizza [37], enabling a monetary value to be attached to BTC for the first time. Back then, Bitcoin’s price stood at less than a penny, while at today’s prices, they would be worth more than 430 million USD. In November 2020, Bitcoin processed around 293,000 daily transactions. By August 2021, 18.7 million bitcoins were still available, which leaves roughly 2.3 million yet to be introduced into circulation, while that last bitcoin will be delivered somewhere in February 2140 [38]. In November 2021, the Bitcoin market capitalization reached over 1148 billion U.S. dollars.

Obviously, there are other cryptocurrencies available on the market, and among the first to emerge were Namecoin (NMC) and Litecoin (LTC). The former is the first cryptocurrency that acts as a decentralized domain name system [39], while the latter is considered as the “silver standard”, becoming the second most accepted crypto by both exchanges and miners [40]. In 2021, it is estimated that there were over 6000 cryptocurrencies [41], with a total capitalization of 1538 billion U.S. dollars (excluding BTC) as of November 2021 [42]. By the end of February 2023, there are over 22 k cryptocurrency projects, with the total value of 1 trillion U.S. dollars, where ten largest cryptocurrencies by market capitalization are: Bitcoin ($452.1 billion), Ethereum ($200.0 billion), Tether ($70.9 billion), Binance Coin ($47.9 billion), U.S. Dollar Coin ($42.4 billion), XRP ($19.3 billion), Cardano ($12.6 billion), Dogecoin ($10.8 billion), Polygon ($10.7 billion), and Binance USD ($10.6 billion) [43].

The skyrocketing growth of the global crypto market value has attracted not only honest investors but also scammers. Generally speaking, cryptocurrency scams fall into two different categories, namely: data breach, and disinformation [42]. By definition, a data breach is a “compromise of security that leads to the accidental or unlawful destruction, loss, alteration, unauthorized disclosure of, or access to protected data transmitted, stored or otherwise processed” [44]. It should be noted here that we will use a more general term later on, namely a security breach, since this naming covers a wider spectrum of objects, including applications, services, networks and hardware devices [45]. The second category of scams concerns disinformation, that is, false or inaccurate information, deliberately spread to deceive or mislead [46]. By nature, intentionally, maliciously deceptive information is created and spread with the aim of tricking cryptocurrency investors, intended to result in financial or personal gain, hereafter termed fraud [47].

With waning trust in local currencies, facilitated by the proliferation of social media, the estimated number of global crypto users passed 106 million in February 2021 [48], which means a rise by more than 881% compared to the past year. The top three reasons are that it is easy to make trades, it is exciting to invest in, and there is potential for high growth in a short period of time [49]. However, these premises have brought about new threats, imposing a significant impact on financial stability for not only the individuals but also for the global economy as well. As security breaches and fraud schemes have become increasingly sophisticated, modern security measures have become less and less efficient, lacking the ability to provide an adequate level of protection.

## 3. Cryptocurrency Wallets

A cryptocurrency (or digital currency) wallet (CW) is an application that generates and stores a pair of cryptocurrency private and public keys [50], facilitating the transfer of funds between individuals [51]. In particular, CWs are used to manage the user’s digital assets, including creating an account address, managing cryptocurrency transactions, supporting queries of transaction records, as well as other basic financial services [52]. Interestingly, digital currency wallets, as may be suggested by the name, do not store digital currencies [53].

The most general classification distinguishes two types of cryptocurrency wallets:

- Custodial wallet in which the private keys are held by a third party organization,
- Non-custodial wallet in which all the blockchain custodian services resides with its user.

Some authors point out that the former may be considered less secure than the latter [54]. Nevertheless, custodian wallets are claimed to be the best entry point for users who lack a technical understanding of blockchain technology, impose less responsibility and are usually more convenient to use [55]. On the other hand, a non-custodial wallet delivers a spectrum of security-based benefits, enabling the user to solely possess the private key with its associated public address. Typically, this takes the form of either a file or “mnemonic phrase” of between 12 and 24 randomly generated words. This feature enables the user to conduct private P2P crypto trades, in particular trades on assets that are not listed on custodial crypto exchanges [56]. Both custodial and non-custodial wallets are available in three different settings: hot, cold, and hybrids (of the two above).

A hot wallet (HW) is always connected to the Internet 100% of the time, allowing a user to send and receive digital assets on demand. However, due to the instant connection, a hot wallet is vulnerable to attack by malware or hacker. Thus, holding a large amount of digital assets in a hot wallet seems to be a poor security practice. Based on the technology used, three different types of HWs are distinguished [57]:

- Desktop wallet (e.g., Atomic Wallet, Eidoo, Exodus) is a piece of software that can be downloaded and installed on a personal computer (desktop, laptop); it is claimed that this scenario offers one of the maximum tiers of security.
- Online wallet (e.g., Coinbase, GateHub, Guarda) is a web-based software application [58], located and executed remotely in a service provider’s cloud environment.
- Mobile wallet (e.g., Edge, Coinomi, Enjin) is a stand-alone application devoted to mobile devices (e.g., smartphones, tablets) [59].

In contrast, a cold (or hardware) wallet (CW) (e.g., Corazon, Keepkey, Sugi) is designed to generate and store a user’s private keys in an offline environment, known as cold storage. They are usually implemented as USB-based plugin devices, which appear to their user as similar to USB drives. A cold wallet, based on an off-line hardware solution secured by a passcode or any other additional authentication means, is claimed to be significantly safer than software-only equivalents [60,61]. A rule of thumb is to use a CW to store a relatively large amount of digital assets, or to make regular savings into crypto as part of an investment portfolio.

Hybrid (Hot-Cold Hybrid, HCH) wallets (e.g., Exodus, Trezor) have emerged as trade-offs, seeking to find a balance between hot and cold wallets, by taking advantage of dual online and offline technologies [62]. In practice, HCHs enable the users to safely store a set amount of assets offline in cold storage, meanwhile also sharing an amount of crypto online for instant trading.

It should also be noted that, despite the digital nature of cryptocurrencies, one can also use paper wallets. Difficult to access and completely off-line, they take the form of printed sheets of paper with public keys and private keys printed out [60], mostly in QR Codes that need to be scanned to be used. In addition to the risk of fire, theft, loss, or water damage, there are other reasons paper wallets have become obsolete. A user must use a trusted wallet generator, but since numerous are open-source software, malicious hackers have developed modified versions available online that can steal the user’s keys [63]. However, paper wallets are considered one of the most hack-proof wallets of all.

To sum up, the security level of the cryptocurrency wallet depends on its type, taking into account the key management schema. It seems rational to use cold wallets since their off-line design effectively protects the stored assets from being stolen [64]. On the other hand, since there is no limit to the number of wallets, one can split their assets across multiple wallets, diversifying them not only by the amount but on the type as well. Furthermore, a user’s password policy should follow best practices, such as minimum password length, complexity and history enforcement, minimum and maximum password age [65]. Another primary concern is the design and scheduling of backup and recovery maintenance plans to respond effectively in the event of data loss [66]. From a user perspective, it seems reasonable to recognize the impact of these issues on the security of cryptocurrency wallets.

## 4. Security Architectures

Security architectures could be defined as global systems essential to protect the IT infrastructures and technologies that are required to construct secure platforms [67]. According to Conrad et al. [68], security architecture is a complex concept that includes security components of software, hardware, and operating systems, as well as procedures that make it possible to build, adjust and evaluate those security components. Beyond this, further essential elements of security architecture include, inter alia, legal regulations, internal processes and procedures [69], integrated with other autonomous physical systems (e.g., fire protection, and anti-theft systems) [70].

When reviewing the existing architectures of digital currencies – the decentralized architecture of bitcoin is worth special attention [71]. A blockchain system and its child—a bitcoin cryptocurrency—are perceived as core digital architectural solutions. Bitcoin is currently the most popular cryptocurrency using blockchain; its architecture reduces the transaction and intermediary steps and costs by eliminating third parties, bank blocks, internal networks and transaction aggregators [33,72]. Based on blockchain, a traditional Bitcoin System of Systems (SoS) architecture is supported by the Bitcoin Network that consists of the Bitcoin Foundation, Bitcoin Payment Processors and e-stores using Systems Modelling Language, with an irreversible history of all Bitcoin transactions that is passed from the payer to the recipient, which makes it possible to verify the real owners of all Bitcoins [72].

Basically, blockchains are nothing more than databases, deployed and managed for the benefit of their owners [73]. In common sense, blockchain is the technology that underpins modern cryptocurrencies. More formally, according to IBM, blockchain is a shared, immutable ledger that facilitates the process of recording transactions and tracking assets in a business network [74]. Transactions confirmed and validated through blockchain are immutable, while a transaction timestamp history is available for the wider blockchain user community, supporting the transparency, traceability and irreversibility of the blockchain technology [75]. The so-called “fingerprint attribute”—the uniqueness of each block in a chain—is fundamental to the blockchain architecture, while malicious attempts to swap blocks of data and modify their hashes would lead to the link breaking and the following blocks becoming invalid [76]. There are cryptocurrencies that are based on Blockchain 1.0 [77], e.g., Bitcoin, Dogecoin and Litecoin. Blockchain 2.0 is used on smart contracts and properties, while Blockchain 3.0 could have more general applications, from healthcare and educational institutions to scientific and governmental projects [78].

Blockchain is perceived as a safely encrypted ledger and a reliable system of cryptocurrency exchange [79,80]. The security of blockchain architecture is enhanced using a procedure called “proof of coinage” [81]. According to [79], in order to ensure cybersecurity and digital currency safety, blockchain technology should be further exploited. One of the possible solutions to preserve data safety and integrity is to utilize the metastable blockchain protocol that ensures greater security of blockchain platforms [82].

According to the World Bank, a distributed ledger refers to a novel and fast-evolving technology for recording and sharing data across multiple data stores, termed as ledgers, which are enablers for transactions and data to be recorded, shared, and synchronized across a distributed network of different network participants [83]. Distributed ledger technology (DLT) seems to be a promising approach, both addressing the limitations of the current digital identity methods [84] and applications deployment [85], at the same time being highly secure, transparent, and tamper-proof [86].

While there are a few high-tech solutions under DLT, such as hash-graph, holochain, tangle, side-chain and blockchain, which differ in terms of consensus algorithms and data storage methods, blockchain is the technology based on which cryptocurrencies arose [87]. In terms of an algorithm allowing a new block creation and mining within blockchain, one can distinguish among: proof of stake (distinctive for EOS, Cardano (ADA) and Tron (TRX) [88]), proof-of-work (used in most popular cryptocurrencies, e.g., Bitcoin (BTC), Litecoin (LTC) and Ethereum (ETH) [89]) and proof-of-capacity mechanism (used in Ripple (XRP) and Signum (SIGNA) [90]). Additionally, proof-of-burn consensus algorithm allows the miners to “burn” coins without extensive energy consumption, thus preventing double-spending (e.g., Slimcoin [91]). All the mentioned algorithms aim at approving and validating transactions, thus ensuring the security and transparency of the respective blockchain [92].

DLT solves the problem of centralized supervision by peer-to-peer verification and multiple—instead of one—data storage locations [93]. Different blockchains can also differ in terms of permission strategies: those could be public, private, consortium, or hybrid ones [94]. Public blockchains are open to all, they are ‘permissionless’, with unlimited access to transactions history and mining, popular public cryptocurrencies examples of which are: Bitcoin, Litecoin, Ethereum, Dogecoin and Monero [95]. Private or ‘permissioned’ ones are restricted to a close smaller and controlled users’ group with additional moderator’s function (e.g., Enterprise, Hyperledger and Ripple) [96]. Finally, hybrid blockchains synthesize both private and public elements, with open history and smart contracts for verification; while consortium or ‘federated’ blockchains involve decentralized chain of users belonging to the organization and managed by pre-established nodes [97]. All those are examples of different blockchains that build up different mining, verification, storage and transactions solutions.

According to [79,81], blockchain technology is considered secure and stable, while its network architecture is changeable. For example, it is seen as a reliable response to the Internet of Things (IoT) security vulnerabilities (which could stem from application layer threats, network layer threats and physical layer threats [98]), through secure data sharing, secure authentication, access, and control of numerous IoT devices, as well as secure data storage [80]. The so-called consensus algorithm of blockchain is aimed at ensuring architecture safety [81]. A classical blockchain consensus protocol is intended to both eliminate possible faults and to guarantee the security of the blockchain [99].

Examples of blockchain security algorithms are corresponding consensus algorithms (e.g., proof-of-stake, delegated proof-of-stake, Raft, proof-of-work and practical Byzantine fault tolerance), which deal with potential distribution system problems (e.g., Byzantine Generals Problem) and are elaborated for different scenarios [81]. For instance, proof-of-work functionality is an algorithm to reach consensus in a network by using real processor cycles to create new blocks of blockchain, thereby verifying and protecting the blockchain history and preventing double-spending [72,100]. The Byzantine Generals Problem is applicable in case of the distributed systems compromise, and could be solved with the complex Paxos algorithm, or the simpler and more popular Raft algorithm [81]. On the other hand, to provide more secure identity management, one can also consider employing the most efficient encryption methods with the aim of optimizing user identity verification time [101].

Bitcoin is an electronic money system based on a reusable proof-of-work (PoW), which uses cryptographic controls with a scarce cryptocurrency supply and irreversible hard transactions performed with no centralized authentication, which provides user anonymity [72]. The traditional blockchain architecture consists of four layers: application layer, extension layer, network layer and data layer. However, the Bitcoin architecture cannot currently ensure perfect privacy protection at such high transaction rates [99]. For example, when the throughput is increased, the Bitcoin protocol is exposed to a double spend attack, as attackers could switch the chain and even replace it with one with a lower processing capacity [102]. Moreover, Bitcoin records do not ensure absolute transactional privacy, since there are methods to link the users’ data (e.g., IP addresses) to their pseudonyms [103]. There are techniques to ensure better privacy of users’ data, such as using a mixing service (e.g., Coinjoin [104]), unlinking transactions and their origins (e.g., Zerocoin [105]), and hiding the coins’ values and amounts (e.g., Zerocash [103,106]).

To maintain the security architecture, three aspects need to be taken into consideration: data privacy and security, which is linked with social aspects; system security—related to technology and computing, as well as operating system security—aimed at counteracting digital fraud [69]. Essentially, the security of each of the systems’ layers (infrastructure, network and application layers) needs to be guaranteed, while another important aspect is to ensure the availability, integrity and confidentiality of information and data [107]. The information security architecture is intended to ensure that the data are encrypted in a protected user device, the users are authenticated, the access is authorized appropriately, the logging is audited, and decryption and safe storage of data are ensured in a securely protected resource [108].

With the growing popularity of so-called Central Bank Digital Currencies, CBDC or digital money used for cross-banks settlements [109], there are endeavors to consider blockchain technology for CBDC purposes. While, according to Zhang and Huang [75], a permissioned blockchain, or a blockchain based on permissions is a better solution for Central Bank Digital Currencies, blockchain’s limitations in terms of, e.g., scale, use scenarios, inter-operations and performance impose certain barriers on the use of the technology for CBDC [110]. Additionally, legal and procedural requirements, and the internal regulations of central banks, prevent the full-scale incorporation of decentralized technology based on anonymity, irreversibility and lack of compliance with external regulations [111].

Digital security is an integral part of the bigger digital infrastructure and network, therefore it should not be set up separately from the users, devices, the network and environment. Moreover, one security architecture cannot be a universal solution to different threat scenarios, which is why a so-called tier-based or reconfigurable security architecture that provides changeable security settings is preferable [112]. More specifically, the reconfiguration mechanism, which is a part of a security architecture, ensures monitoring of various characteristics to dynamically react and activate those security mechanisms that are more appropriate in a particular situation [113]. Additionally, a reconfigurable security architecture is a dynamic solution that is able to localize and detect cyberattacks, therefore ensuring the actual security warranty [114].

To sum up, the security architectures of digital currencies proposed by a wide range of research concentrate on blockchain technology, and especially on Bitcoin solutions, which are perceived as relatively secure, due to the reliable data access, storage, encryption and transfer arising from the blockchain architecture itself. However, security threats related to blockchain systems come from networks, software, and hardware fragility, as well as human factors. Potential solutions aimed at increasing blockchain security are the corresponding consensus algorithms, meta-stable blockchain protocol, external-internal security trade-off and more reliable network and software.

## 5. Security Models

In general, security models are used to define the notion of security embodied by a computer system [115]. McLean points out that security models have been applied to “describe any formal statement of a system’s confidentiality, availability, or integrity requirements” [116]. In other words, three core ingredients, namely confidentiality, integrity, and availability, build up the CIA triad model [117], which is now widely recognized and typically adopted to address principal information security objectives [118].

The definition of confidentiality states that it is “the process of and obligation to keep a transaction, documents, etc., private and secret,” or, in a more narrow sense, it is “the right to withhold information from others” [119]. In the context of cybersecurity, privacy means the freedom from damaging publicity, secret surveillance, public scrutiny, and any unauthorized disclosure of the user’s personal data or information [120], while secrecy is the practice of maintaining privacy [121].

Thinking in the categories of a cryptocurrency system that requires the generation of cryptographic keys and seeds, a user needs to pay close attention to preserve their own privacy. On the other hand, having unguessable numbers obviously provides the first line of defense against unauthorized access [122], but even more importantly, protects against unwanted actors impersonating the intended key (seed) holder [123]. Therefore, to mitigate risks concerning the unintentional disclosure of the wallet-holder’s identity or stolen keys, one should follow best practices such as using keys (seeds) only in trusted environments and requesting a minimum of two signatures for performing transactions. It is worth noting here that the aforementioned fraudulent incident of Mt. Gox is believed to have occurred because the company involved did not use a multi-signature approach to store the private keys of the wallet-holders [124].

In the realm of security, integrity refers to the accuracy, consistency, and completeness of data [125]. Here, however, one question arises: what is the meaning of these three notions? First, accuracy is strictly related to the notion of the magnitude of an error [126]. Second, consistency is defined as the absence of any discrepancy between particular data values concerning the same object [127]; typically, data consistency is considered under three different dimensions [128]:

- Point-in-time consistency means that data is said to be point-in-time consistent if all related data is the same at any given instant in time.
- Transaction consistency means that the data must be in a consistent state before and after a single transaction is executed; if an error occurs, all submitted changes are rolled back and the data returns to the original state.
- Application consistency refers to the state in which all intra- and inter-related data are synchronized and represent the true status of applications.

For cybersecurity of cryptocurrencies, the blockchain is typically applied to systems that require both immutability and integrity checks [129]. By design, blockchain-based systems eliminate the requirement of a third-party trusted authority. Instead, to preserve the consistency and reliability of both the data stored and transactions performed, blockchain adopts a decentralized consensus mechanism and cryptographic security measures [130]. A consortium of multiple organizations can share the responsibilities of maintaining such a system [74].

However, a common misconception is that the use of a blockchain alone can ensure data integrity [131]. By defition, the data integrity involves preserving the accuracy, reliability and stability of data [132]. Even though blockchain has the capability of reliably preventing an undetected data modification once it has been confirmed on-chain, it will only enforce this mechanism on successfully-input data. In other words, if the data is not accurate at the time of input, then putting it on a blockchain does not benefit in any way, except in preserving its immutability. Unarguably, the old saying “garbage in, garbage out” is also valid here. Hence, defining and applying a data hygiene action plan is a critical precursor to any blockchain deployment, but this is still claimed by some to be hard to achieve in immutable settings [133].

From a user perspective, best practices regarding data integrity might concern the following precautions: (*i*) generating unique addresses for every transaction, (*ii*) checking identification, background of all key (seed) holders, and their references, (*iii*) storing keys which have signing authority in different locations. In fact, the blockchain inherently shifts all the integrity responsibility to the user since there are no internal auditing routines regarding data checks against errors, fraud, illegal acts or key losses. Regarding the last problem mentioned, if the cryptographic keys are compromised, the identity of the individual (or any other entity) is lost, and can be abused in many ways, potentially resulting in considerable damage [134].

Moreover, security policies such as a separation of duty (SoD) [135] and the principle of least privilege (PoLP) [136], as well as internal audits, and external audits [137] including also governmental bodies (e.g., Internal Revenue Service (IRS) [138]), offer assurances to the investors, shareholders and owners.

The last ingredient of the CIA model is availability. The general notion states that availability means the quality or state of being easy or possible to obtain, or being ready for use [139,140]. While the above definition seems not to be difficult or elusive to understand, its explication takes different forms in the computer science and other related disciplines [141]. Having said that, below, we provide only some of its definitions, but those that are widely recognized and referred by both theory and practice.

- In the context of the criteria for evaluating computer security provided in the Information Technology Security Evaluation Criteria (ITSEC), availability means prevention of the unauthorized withholding of information or resources [142].
- In the context of the fundamental objectives of information security defined in the Federal Information Security Management Act (FISMA) availability aims at ensuring the timely and reliable access to and use of information [143].
- Along with integrity and confidentiality as the basic security properties and the targets of security threats, availability is the ability of a system to ensure that an asset can be used by any authorized parties [144].

However, if one carefully analyzes the above definitions, there is common ground of understanding and, in fact, strong agreement underlying the written discrepancies between the seemingly incompatible views on availability. To conclude, availability means that information is promptly accessible for only authorized users. Nevertheless, expectations formulated toward availability are far-reaching, borrowing its qualities from non-functional requirements such as capacity, performance, usability and fault tolerance [144]. Yet, in this case, such a broad view hardly helps to conceptualize its concise meaning.

Considering availability in terms of the above-formulated definition, two paradigms come to the fore, namely reliability and access control. While the former can be defined as the probability of a cryptocurrency service (an app) to meet certain performance requirements, the latter can be specified as the means to control privileges or rights to cryptocurrency assets. In practice, availability can be measured by the percentage of the availability of a service (or app) for its users, or even simpler, it can be expressed by the duration of service unavailability in a fixed period of time (e.g., week, month, year), usually termed downtime [145].

Actually, access control is a major part of any system’s security [146], typically imperative for these responsible for managing financial assets [147]. The access control is governed by the security policies [148], which precisely define the authorized actions for all users in the scope of a particular wallet, including managing the encryption keys used to digitally sign transactions, and buy and sell cryptocurrencies. However, it should be noted that current access control methods, static by nature, might be inadequate for next-generation systems [149].

Last but not least, apart from its prominent role in information security practice [150], voices of criticism against the CIA model have emerged on multiple occasions [151,152,153,154]. Indeed, the orientation of the model is, by design, narrowed to technology, and, as a consequence, its adoption intelligibly leads to the organizational and social aspects of security being overlooked. While the greatest risk involved investing in cryptocurrencies lies within the increasing number of crypto scams on people [155], then the arguments for re-examination and reorientation of the CIA model seem to be rational and eventually convincing.

## 6. Secure Data Transmission Methods

Blockchain technology is recognized as the dominant technology in the cryptocurrency (and digital currency) world market. Not only the largest, currently leading cryptocurrencies such as Bitcoin and Ethereum are implemented with this technology, but many other popular currencies are also blockchain-based (Litecoin, Deuterium, etc.).

Blockchain technology has been adopted for many different fields in which there is a need to exchange some valuable assets. Most notably, there are blockchain-based solution proposed for smart grids [156,157], healthcare and telemedicine [158], smart insurance [159], vehicular energy networks [160], databases [158,161], cloud computing [162], software-defined networks [163], wireless sensor networks [164], trading energy contracts [165] and livestream video transmissions [166]. Some of these solutions use existing implementations of public blockchains, such as Bitcoin or Ethereum, and some use their own systems. Most of these systems, however, need to provide some sort of currency exchange mechanism which is highly secure. The security model to be applied for the data transmission of digital currency most often depends on the field in which the currency will be applied. In terms of scope and permission level, blockchains can be divided into three different types [166]:

- public type—a public blockchain that every Internet user can operate with (Bitcoin, Ethereum, Litecoin, Deuterium, etc.),
- private type—a blockchain that is a private property of an organization; there is an actor (administrator) who gives permission to other users to access data in order to operate with the blockchain,
- consortium (federated) type—a field of companies, organizations, individuals, representatives or agents together make the decisions regarding the blockchain network; verification of transactions and blocks is implemented through different centers, which decreases the number of points of failure.

Blockchain was recognized as a disruptive technology by offering data immutability, security, decentralization and transparency [158]. It also ensures data integrity, data ownership, and a trusted data source [158]. The peer-to-peer nature of the transactions in blockchain-based systems bypasses third parties’ participation in the process, which eliminates the single-point-of-failure problem [157], could positively affect user anonymity, protecting their privacy [158], and could lead to an overall less expensive system [157].

The hash function used by a blockchain should be one-way (i.e., it is hard to determine the input string from the output hash), and collision-resistant (no two inputs can ever produce the same hash output) [167]. Several different hashing algorithms are used in popular blockchain-based systems, for example [167]:

- Bitcoin uses SHA-256,
- Ethereum uses Keccak256,
- Litecoin uses Scrypt,
- Dogecoin uses Scrypt.

Hashes of blocks are stored in a data structure called a Merkle Tree (or hash tree [162,167], introduced by Ralph C. Merkle [168]. The Merkle Tree is a tree data structure in which hashes of data blocks are stored in leaves, and every non-leaf vertex stores the hash of its children content (hashes). A Merkle Tree is usually implemented as a binary tree (every non-leaf vertex has at most 2 children). The hash stored in a Merkle Tree root (Merkle root) is then stored in the header of a data block, and can be used to verify that the transmitted block is whole, undamaged and unaltered. Public and private key pairs are often generated using the Elliptic Curve Digital Signature Algorithm (ECDSA) or RSA.

There are some reports of security issues that are still present in data transmission in blockchain-based solutions. However, solutions or countermeasures have already been proposed by researchers for many of these challenges in the form of modifications to blockchain algorithms and data structures. Another important issue is collision resistance of cryptographic hash functions [169].

Solutions based on popular public blockchains, such as Bitcoin or Ethereum, are not applicable for transactions with huge volumes of data due to scalability issues, which could be mitigated with technologies such as the InterPlanetary File System [170] and BigChainDB [171]. In public blockchains, there are also problems with user privacy, which can be eliminated by implementing a private or consortium-type of blockchain with a hybrid encryption method, in which the user’s data is encrypted with a symmetric password, and afterwards the symmetric password is encrypted with an asymmetric pair of keys [158]. The anonymity of the user can be further secured by implementing Zero Knowledge Proof protocols for authentication [172].

The long distance in kilometers between trading locations is a characteristic feature of the current globalization of transactions. Long transmission distances (often implemented via satellite transmission systems) can adversely affect the security of real-time transactions [173]. To combat this, several optimizations for hash algorithms have been formulated [173].

It is worth mentioning that digital currencies not based on blockchain often offer significantly faster transaction speeds. For example, Ripple (XRP) confirms its transactions in around 5 s, while it takes approximately 10 min to confirm a transaction in Bitcoin [174]. There are studies and experiments performed to optimize other cryptographic operations in cryptocurrencies as well, including secure key transmission and smart contract execution [175] and the process of cryptocurrency mining [27]. These optimizations not only speed up the process, but often also minimize the amount of energy needed for computations, which is crucial for smart grids [176,177].

Some researchers propose secure validation methods and pricing schemes for blockchain-based peer-to-peer applications with a game theory approach [178]. This idea promotes the idea of rewarding users that are helping with a successful delivery, prevents selfish actions exhibited by users, and prevents their collusion.

To sum up, blockchain technology is commonly used for digital currencies nowadays because of its high level of security. The idea is relatively new, but quite popular among researchers around the world, who are proposing modifications to the original idea to overcome the increasingly many challenges identified (i.e., high energy consumption, long operation times, scalability issues, etc.) and security issues (weaknesses of internally used algorithmic procedures, increased vulnerability to attacks caused by long-distance transmissions, and problems with users’ privacy and lack of anonymity, just to name a few).

## 7. Social Engineering Attacks and Countermeasures

Naturally, the users of cryptocurrencies are at risks of scams and identity theft. In general, social engineering techniques take advantage of deception and manipulation [179]. In place of attacks on software and hardware technologies, social engineers target humans, aiming to compromise private information. In 2022, Hetler specified nine common cryptocurrency scams, namely: Bitcoin investment scheme, employment offers and fraudulent employees, fake cryptocurrency exchanges, man-in-the-middle attack, phishing scams, ponzi schemes, romance scams, rug pull scams, and social media cryptocurrency giveaway scams [180]. They often leads to theft or distortion, data destruction, or fake transactions [181]. Yet, due to their unconventional and sophisticated nature, social engineering attacks (SEAs) are still being heavily investigated [182], in order to better understand their mechanisms of occurrence and scenarios of performance, which is essential to prevent and reduce their negative impact.

### 7.1. Social Engineering Attacks

By definition, social engineering is “the use of deception to manipulate individuals into divulging confidential or personal information that may be used for fraudulent purposes” [183]. The social engineering attack (SEA) is defined as an action, where “an attacker uses human interaction (social skills) to obtain or compromise information about an organization or its computer systems” [184]. Social engineering is targeting Internet-related systems in particular, and is increasingly being applied to cryptocurrency users [185].

From a user perspective, a security breach involves stealing passwords and wallet private keys in the case of cold (off-line) wallets [186], or obtaining unauthorized access to the user’s on-line accounts, including information such as an email address with the password, and phone number linked to the account, as well as access to the associated email account in the case of hot (on-line) wallets [187]. Such an attack can be performed by spreading fake news, which is defined as “purposefully crafted, sensational, emotionally charged, misleading or totally fabricated information that mimics the form of mainstream news” [188].

One can distinguish three categories of SEAs [189]:

- technology-based,
- human-based (social approach),
- hybrid (socio-technical approach).

#### 7.1.1. Technology-Based Attacks

Using technical tactics, the social engineer employs computer applications to trick users into taking a specific action. People-based tactics, on the other hand, are performed by attackers who understand the shortcomings of the human psyche [190].

Hackers use many different techniques to steal a user’s sensitive information, and thus, for example, gain unlimited access to their bank account.

- Spyware. Spyware is very difficult to detect. Its task is to discreetly collect and send other people information about the user, such as personal data, payment card numbers, access passwords, addresses of visited websites, interests (which can be inferred from the search queries) and e-mail addresses. Such a program is usually associated with another application, or a file downloaded from a website on the web. Sometimes, it is also attached to e-mail attachments [191,192].
- Adware. These types of programs, also known as adware, are very annoying, but usually not particularly dangerous. They work by displaying pop-up ads both when running other applications and when idle. Similarly to spyware, adware is most often bundled with free programs downloaded from the web [193].
- Keylogger. This software records the keys pressed by the user and thus collects data such as credit card numbers and passwords. Keyloggers also come in the form of small devices attached to the keyboard port [194].
- Ransomware. Ransomware is a much more advanced cyberattack technique, which consists in blocking access to certain files and offering to unblock them for a hefty fee. Of course, hackers rarely keep their promise, even if they receive the ransom. Such a program is typically installed simultaneously with other programs without the user’s knowledge while using an unsecured network, infected website, or email attachment [195].
- Trojan. A Trojan (Trojan horse), is a program that imitates a useful application that the user installs on their device. This software gives unauthorized persons access to the computer or telephone. Similarly to other types of viruses, the Trojan can hide in email attachments, illegally downloaded movies, and free applications [196].
- Worm. These types of programs have the ability to replicate and spread by themselves using a computer network. They are usually used for activities such as sending e-mails or destroying files on the disk. Such activities consume the bandwidth of networks and devices, making the latter often become very slow and even stop responding to commands [193].

#### 7.1.2. Human-Based Attacks

Social engineering attacks on cryptocurrency users exploit the human factor [185]. Socially based cyberattacks can appear by employing various acts, such as tailgating, impersonating, eavesdropping, shoulder surfing, dumpster diving, reverse social engineering, and others [197]. Attackers often use the five principles of persuasion: first—authority and power; second—social proof, liking, likeness; third—deception; fourth—commitment, reciprocity and consistency; and fifth—distraction [198].

- Impersonating. Through impersonation, the threatening player assumes a false identity to gain credibility that will enable them to perform malicious acts such as piggybacking, pretexts and quid pro quo.
- Tailgating/piggybacking. Tailgating, another popular social engineering program, involves following someone with authorized access into a building or system and thus using someone else’s authorization to gain access to a data source. This is similar to pretending to be someone who has forgotten an ID, supposedly in need of help and playing on the innate human trait of being helpful [199,200]. Tailgating is the act of following the unconscious goal of a person with legal access through a secure door into a confined space. This can be compared to when the attacker asks the victim to hold the door, or simply walks in before it closes [197].
- Eavesdropping. Eavesdropping is an act of secretly or stealthily extracting information from an interaction in which it is taking no part, including channels such as emails, instant message, videoconference and phone lines [197,201].
- Shoulder surfing. Through shoulder surfing, an attacker directly observes the victim over their shoulder to collect personal information and credentials [197,201].
- Reverse social engineering. The attacker encourages their victim to initiate the interaction. The player lurks, plays the role of a trustworthy character, fabricates a problem for the victim and indirectly presents a real solution. They inspire trust and extort the data they need [197,201].
- Pretexting. Malicious hackers pretend to be someone other than who they are, such as a system operator, to obtain confidential information about a person or company. For example, an attacker calls an employee and asks them to confirm their username and password for security reasons [182,190]. Using a variety of pretexts and deception, a hacker can create a fake website on the Internet (such as a fake bank website) to influence a targeted victim to disclose confidential information to perform an action that poses a threat to themself or their company [202].
- Quid pro quo. The main feature of this type of attack is to give someone something back. The attacker does a good deed for the victim, who may then be more likely to return the favor. The easiest way to prepare for an attack is to search the Internet and gather information about the company. It is also possible to call to obtain specific information and to exploit published vulnerabilities [189,199,203].
- Dumpster Diving. During dumpster diving, attackers search corporate computer trash cans, assuming they will find useful protected information about the company, network, and its employees [185,204]. Dumpster diving is a non-traditional search and is legal and very common, and often provides a wealth of information [205].

#### 7.1.3. Hybrid Attacks

The following types of hybrid attacks have been identified, using social influence techniques (the so-called socio-technical approach):

- Baiting is an example of a social engineering attack based on malware-infected media storage made to appear abandoned in a public place, to be found and used by a future attack victim. For example, a USB device with an appealing label infected with a Trojan horse could be left in a bank location or another place with an increased probability to be found by a targeted victim [206]. Hackers preload malware onto external storage devices (e.g., CDs or USBs) and strategically leave them in generally accessible public areas of the targeted company. When employees pick up the CDs or USBs carrying the malware, they connect them to their computers [190,207].
- Trolling is a form of cyberbullying and harassment on the Internet; its manifestations include, for example, publishing and sending information or videos of public suicide attempts, songs, such as lullabies for children, to which hackers attach malware [207,208]. Trolls manipulate public opinion to spark social discourse and exploit “human bias against binary choices” [209]. The tactics used by trolls to achieve the desired extremes are “lies, evasions, untruths, alternatives, improbable theories, distortions, ad hominem attacks, and other rhetorical measures as part of Machiavellian propaganda or handover campaigns” [209]. Trolling uses phishing attack methods, computers, and network systems to manipulate Internet users’ perceptions of information, make them think differently, and motivate them to do something they would not have thought of on their own.
- Phishing is a form of attack in which social engineers send fake email messages that recipients find legitimate. The email may ask you to click on a malicious link or take action that exposes sensitive data [190,210]. A phishing attack is fraudulent activity and a crime that is aimed at acquiring personal information, e.g., personal ID details, credit card and bank details, such as passwords and phone details, by pretending to be a legitimate entity or person with a pseudo-legitimate purpose [211].
- Pharming attack is a domain name system (DNS)-based phishing attack that relies on tampering with bank host files or DNS [212]. In a DNS-based phishing attack, a hacker redirects the user to a fraudulent website or the hacker’s device when the attack victim tries to access a legitimate bank website, in order to obtain a copy of the user’s bank credentials [212]. A pharming attack can be performed by a malware installation on the bank user’s device or by tampering with the e-bank domain; in any case, when entering the proper bank URLs on the browser, the user is automatically redirected to a fraudulent web page [213].
- Malware attachments Phishing also often contains malware attachments or programs that attackers install on the user’s device. Malware-based phishing could take place when the bank user or employee accesses an unauthorized webpage and unintentionally downloads a malicious piece of software [212]. When the user accesses the unauthorized website, a program with a keylogger is automatically downloaded and installed on the user’s device, which is then used by the attackers to steal confidential information and the user’s bank credentials [214]. Thereafter, the keylogger gathers the user’s personal data and credentials in the form of keystroke information, and sends them to the hackers in a file that will later be used by the hackers to commit financial crimes [214].
- Watering Hole is an attack that requires advanced technical knowledge. The attacker identifies one or more legitimate websites regularly visited by the targeted user. The hacker looks for vulnerabilities, infects the most vulnerable website, and waits [197,215].
- Smishing is a combined form of SMS and phishing in which attackers send the victim SMS messages containing malicious content. This content sometimes contains links that redirect the user to websites with malicious applications and user interfaces [216].
- Whaling is a type of attack which specifically targets top management, profiling company goals using highly personalized threat analysis. These forms represent broad categories and there is a need to develop clearer descriptions and details of specific attacks in order to understand their rate of occurrence and their impact on organizations [217].

In summary, cybercriminal activities are currently targeted at cryptocurrencies due to the pseudonymity and privacy they offer. Attackers continue to cause new losses, even as masses of scientists are actively analyzing and developing innovative defense mechanisms to prevent these actions [218]. Thus, the most commonly employed attacks are phishing, smishing, and vishing [219]. Phishing attacks are among the most widespread social engineering attacks and can use complex techniques such as, for example, the “Man in the middle” (MITM) attack [212]. The MITM attack is characterized by hackers placing themselves in the middle of the digital communication chain between the e-bank and its customers, where both the bank and the customer are not aware of the attack, while confidential data and credentials are compromised [220]. Regardless of the chosen attack technique, the hackers aim to gain e-banking users’ data and credentials in order to conduct financial frauds and illegally harvest the users’ money for the hacker’s benefit [212].

### 7.2. Countermeasures against Cyber Attacks

Regardless of the social engineering method (see Table 1, in order to counter the attack, bank users and staff should regularly complete online security training, be aware of the potential threats and attack techniques, use two-factor authentication, install and upgrade their antivirus software from a legitimate source, and be conscious of the potential threats and suspicious communications/websites they could be exposed to.

The owners of cryptocurrencies definitely have to reckon with cyberattacks of various types. However, regardless of the type of attack, the victim’s trust, naivety, lack of vigilance, lack of knowledge, unbelief in the possibility of an attack, or some thoughtlessness may be to their detriment.

## 8. Discussion

In recent years, researchers have developed various methods to counter phishing. However, the problem still exists [221]. Many users do not take cyberattacks seriously. Cybercrime should be treated the same as any other type of crime, and make it not pay for hackers to attack. Typically, in the case of a cyberattack, everyone focuses on blaming the victims instead of prosecuting the perpetrators. Instead, the companies attacked are treated as the culprits. At the same time, it is accepted that criminals escape punishment due to the lack of a globally agreed legal framework and an adequate justice system [222]. Internet users are reasonably aware of cyber threats but use only minimal protective measures that are usually relatively common and straightforward. Higher cyber awareness depends on a person’s level of cyber-education, competence and knowledge [223] and on the user’s country and the country’s educational conditions [224] as well as their gender [225]. Awareness is also related to the use of protection tools but not to the information that IT users were willing to disclose [226].

In information security research, personality traits are considered primary predictors of human behavior. For example, the so-called Big Five Model identifies five components of personality: agreeableness, conscientiousness, extraversion, openness, and stress tolerance. A user’s confidence, competence, motivation, and previous experience with cybercrime are essential in explaining the impact of the Big Five personality traits on vulnerability to cyberattacks in social network settings [227]. Conscientiousness, agreeableness, and neuroticism strongly reduce users’ vulnerability to cyberattacks in social network settings. While extraversion turns out to significantly increase a user’s likelihood of falling victim to cyberattacks [228].

Personality is the most critical factor affecting, for instance, the susceptibility to phishing. Despite having knowledge and experience, when people encounter something new, their personality strongly influences their behavior. The second most crucial factor is cognitive processing, which shows how a person processes information and affects whether they click on links; some people are more cautious, while others are more casual. The third most important factor is computer knowledge, which can help people better distinguish between phishing and legitimate e-mails [229,230].

Influential cybersecurity professionals who can defend themselves against cyberattacks differ from other employees, even standard information technology professionals, on trust, intellect, sympathy, vulnerability, self-consciousness, assertiveness, and adventure at the trait level [231]. Cybersecurity professionals score significantly lower than other employees in agreeableness, openness, and trust [231,232].

Given the need for cybersecurity specialists to protect their companies and loved ones from outside threats, it is understandable that they may be less trusting of individuals, as anyone can access a computer and pose a threat. Cybersecurity specialists scored higher than other employees on intellect. High correlations were found between information technology specialists and openness, but because intellect is derived from openness, cybersecurity specialists were already inclined to score relatively high on this trait [231,232].

Companies paying ransoms to recover data are signaling to cybercriminals that ransomware attacks are a way to make easy money and encouraging them to continue their criminal activities [233]. Once victims stop paying, ransomware attacks will become less frequent as they lose effectiveness [234]. Even though companies affected by cybercrime are victims, they should protect any data they use, process, and store [235]. Paying cybercriminals to restore access to systems cannot be considered a defense strategy [236], since it does not work in the long run [237].

Building a cybersecurity culture framework with a clear focus on the human factor is essential, which can help detect possible threats from both malicious and unintentional insiders [238]. While the law does not fully protect us from cybercrime, primal human survival instinct tells us that we should defend ourselves [239]. This requires taking a few basic steps. First, every company should employ a dedicated IT security manager, working on-site, with regular contact with company management and the authorities to take security initiatives. Smaller companies also need a person in charge of cyber security who specializes in data protection.

Second, companies must observe digital hygiene. This includes, in particular, mandatory training for all employees so they can detect potential attacks, know whom to report them to, and understand why this is so important. The more employees are involved in implementing digital hygiene, the more aware they will be of the risks and the more effectively they will prevent them [240].

Third, both individuals and teams should receive coaching and training that can strengthen not only their hard competencies in cyber defense. We also suggest the development of individual dispositions and soft competencies in terms of calculated trust and caution, especially to proposals for so-called big wins, conducting business and phishing.

Summing up, such factors as technical and programmatic safeguards at the organization level, team education on cyberattacks and how to defend against them, and individual education and competence development in knowledge, skills, soft dispositions and social skills to defend against cyberattacks can lead to the effective defense against cyberattacks, and stability for the organization.

### 8.1. Theoretical Implications

The theoretical implications of our research are the opportunities to develop conceptual and empirical models based on the issues classified, defined, and analyzed. Our paper provides a theoretical basis for a broad discourse on resistance to the cyber security of digital currencies from both technical and human-oriented perspectives.

### 8.2. Practical Implications

Our research not only contributes to the theory but also provides important practical implications. Our findings can serve as a warning to individual Internet users, as well as companies, organizations, and even local and central governments on how to secure their information systems against cyberattacks. We placed special emphasis on aspects that take into account cryptocurrencies, which, to the best of our knowledge, might become the basis for the exchange of goods and services in the near future.

### 8.3. Study Contributions

Our contribution is a broad critical literature review, the discussion on the background of the development of cryptocurrencies, the review of crypto wallet definitions and classification, the analysis of security architectures with the description and exemplification of the related models. Moreover, we recognized and localized the data transmission methods developed for blockchain-based solutions. Furthermore, we elaborated on the adopted social engineering attacks and adapted countermeasures for cryptocurrencies. Finally, we concluded the paper with the theoretical and practical implementations of the performed study.

### 8.4. Study Limitations

The limitation of our study is that it is only a critical analysis of the literature, it does not constitute an empirical study based, for example, on questionnaires among Internet users. We will address this problem with experimental research in the future, where we intend to target two groups of Internet users: those who have been the victim of a cyberattack and have suffered heavy losses as a result, and those who have been able to resist cyberattacks. We also want to test the psychological characteristics of these two groups of people using the relevant tools, to better suggest to users what qualities they need to develop in themselves in order not to succumb to cyberattacks. We also want to indicate how to effectively defend against cyberattacks from both the technical and cyber perspectives.

## 9. Conclusions

In this study, we have analyzed and reviewed the recent literature on the security of cryptocurrencies, in particular focusing on the both technology-oriented solutions and human-related factors. It seems that neither the former is robust enough nor latter is mature enough to conclude that security issues are no longer present. In fact, on the contrary, a recent report from Trail of Bits provides examples of how immutability of distributed ledger technology (DLT) can be broken by subverting the properties of a blockchain’s implementations, networking, and consensus protocol [241]. On the other hand, people are still the weakest link in the security chain and are chronically responsible for 95% of failures of security systems [242]. Considering the possible countermeasures to implement, obviously one concerns human factor and involves users’ education and training, whereas the opposite relies on the software systems and tools, recently also armed with artificial intelligence-based defense techniques [243].

Nevertheless, the success of cryptocurrencies has brought the attention of governments and central banks. According to the International Monetary Fund (IMF), the interest in exploring the possibilities of launching a central bank digital currency (CBDC) is a matter of the highest urgency [244]. At the moment, 105 countries, representing over 95 percent of global GDP, are exploring a CBDC, while 50 countries are in an advanced phase of exploration (development, pilot, or launch) [245]. In particular, 19 countries from the G20 (Group of Twenty) are considering issuing CBDCs, and the majority are beyond the research stage. Therefore, concerns about cybersecurity and privacy are now matters of state.

At the moment, there are three main varieties of digital currency, namely: cryptocurrency, stablecoins and central bank digital currency. In this realm, security is still a major tenet, including protection against double-spending, counterfeiting, and account and data breaches [246], just to name a few. Undeniably, the desire to come to grips with cybersecurity risks and to be able to find a fair balance for all interested parties has become an area of interest both academically and commercially in recent years, primarily as a consequence of the ongoing revolution [247].

Undoubtedly, new payment systems, with recent technological advancements, will benefit both businesses and individuals in the areas of trust, regulatory stability, and audit transparency [248]. Moreover, the systematic development of users’ security awareness, achieved through education, training and testing, will also provide proactive measures to mitigate the risks and threats. Having said that, in our opinion, future research should pay more attention to elaborating proactive cybersecurity risk mitigation strategies, covering prevention, detection and remediation issues.

## Figures and Tables

**Table 1:** Common social engineering attacks and countermeasures. Own elaboration based on [213].

Type of attack	Countermeasures
Phishing attack	Users should precisely inspect URLs and check whether they redirect to new and suspicious web page, whether emails contains hyperlinks and suspicious attachments. Users should check for spelling mistakes, salutations, pay attention to tones of urgency and emotional intensity of the emails.
Watering Hole Attack	Users should have security proxy gateways able to defend from opportunistic drive-by downloads, prevent criminal redirection, rootkits and malware from being deployed. Users should choose email solution which could perform dynamic malware analysis of the emails.
Smishing attack	Users should avoid unknown and suspicious phone numbers and avoid giving personal information through text-messages. Users should pay attention to tones of urgency and emotional intensity of the message. Users should check suspicious links, applications and campaigns independently from sms or call messages.
Vishing attack	User could ask for the caller’s official credentials and check them and the number independently. Users could avoid answering to unknown and suspicious phone numbers and avoid to give personal information by phone. User could end suspicions call and call the institution back using known and verified number.
Whaling	Users should deploy anti-phishing software performing URL screening and link validation. Users should carefully check the sender email address and credentials. User’s security training is important.
Pharming	Effective and regularly-updated antivirus software is essential to spot pharming attacks. Users should log-in and provide their credentials on https websites that are protected. Users should check security upgrades from a trusted Internet Service Provider.

## Data Availability

Not applicable.
